# Pathways of *Leymus chinensis* Individual Aboveground Biomass Decline in Natural Semiarid Grassland Induced by Overgrazing: A Study at the Plant Functional Trait Scale

**DOI:** 10.1371/journal.pone.0124443

**Published:** 2015-05-05

**Authors:** Xiliang Li, Zhiying Liu, Zhen Wang, Xinhong Wu, Xinle Li, Jing Hu, Hongxiao Shi, Fenghui Guo, Yong Zhang, Xiangyang Hou

**Affiliations:** 1 Institute of Grassland Research, Chinese Academy of Agricultural Sciences, Ministry of Agriculture Key Laboratory of Grassland Resources and Utilization, Hohhot, China; 2 Graduate School of Chinese Academy of Agricultural Sciences, Beijing, China; 3 Experimental Center of Desert Forestry, China Academy of Forestry, Dengkou, China; Henan Agricultural University, CHINA

## Abstract

Natural grassland productivity, which is based on an individual plant’s aboveground biomass (AB) and its interaction with herbivores, can obviously affect terrestrial ecosystem services and the grassland’s agricultural production. As plant traits have been linked to both AB and ecosystem success, they may provide a useful approach to understand the changes in individual plants and grassland productivity in response to grazing on a generic level. Unfortunately, the current lack of studies on how plant traits affect AB affected by herbivores leaves a major gap in our understanding of the mechanism of grassland productivity decline. This study, therefore, aims to analyze the paths of overgrazing-induced decline in the individual AB of *Leymus chinensis* (the dominant species of meadow-steppe grassland in northern China) on a plant functional trait scale. Using a paired-sampling approach, we compared the differences in the functional traits of *L*. *chinensis* in long-term grazing-excluded and experimental grazing grassland plots over a continuous period of approximately 20 years (located in meadow steppe lands in Hailar, Inner Mongolia, China). We found a highly significant decline in the individual height and biomass (leaf, stem, and the whole plant) of *L*. *chinensis* as a result of overgrazing. Biomass allocation and leaf mass per unit area were significantly affected by the variation in individual size. Grazing clearly enhanced the sensitivity of the leaf-to-stem biomass ratio in response to variation in individual size. Moreover, using a method of standardized major axis estimation, we found that the biomass in the leaves, stems, and the plant as a whole had highly significant allometric scaling with various functional traits. Also, the slopes of the allometric equations of these relationships were significantly altered by grazing. Therefore, a clear implication of this is that grazing promotes an asymmetrical response of different plant functional traits to variation in individual plant size, which influences biomass indirectly. Furthermore, we detected paths of individual AB decline in *L*. *chinensis* induced by grazing by fitting to a structural equation model. These results indicate that grazing causes AB decline primarily through a ‘bottom-up’ effect on plant height and stem traits. However, leaf traits, via the process of allometric scaling, affect plant AB indirectly.

## Introduction

An understanding of the main forces driving the decline in individual plant aboveground biomass (AB) induced by overgrazing in natural grassland is of great theoretical interest. It helps us to understand the processes involved in the grazing mechanism and their effect on the grassland ecosystem. It is also of practical importance in predicting the effect of human-induced vegetation productivity decline, biodiversity loss, and rapid depletion of natural resources [1, 2]. A growing body of empirical evidence suggests that the response of plant functional traits (PFT) of an ecosystem can be derived many obvious cascade reactions in the scales of population, community, and ecosystem [3]. Flynn et al. [4] found that PFT can be valuable predictors of the effect of biodiversity on ecosystem functioning. And PFT provide a strong mechanistic foundation for understanding the structure and dynamics of ecological communities [5]. Moreover, it may be that it is an important mechanism of AB change in grassland plants that responds to grazing which is influenced by PFTs. Unfortunately, the current lack of studies on how plant traits affect AB affected by herbivores leaves a major gap in our understanding of the mechanism of grassland productivity decline.

Grazing, as a fundamental and global method of use of natural semiarid grassland, can have a profound impact on ecosystem productivity, biodiversity, nutrient cycling, etc. [6]. In general, grasslands occupy about 40.5% of the world’s land area (excluding Antarctica and Greenland) and support the livelihoods of approximately 1 billion people [7]. However, in the last 50 years, many of these grasslands (and especially the northern grasslands of China in Inner Mongolia) have been in a degraded state. This has affected, in addition to productivity, many vital environmental services such as the hydrology, biodiversity, and carbon cycles of ecosystems [8]. Numerous studies have reported the mechanisms responsible for grassland degradation from the point of view of (or on levels corresponding to) the landscape, ecosystem, community, population, and plant traits [9, 10]. Human activity, mainly in connection with grazing, was found to be the primary reason for grassland degradation [10]. In addition, A growing body of empirical evidence also suggests that an important mechanism maintaining the grassland’s soil-vegetation system is the interaction between various kinds of constructive factors, e.g. presence of living plants, ground litter, soil, etc. [1, 11, 12]. Accordingly, the pattern of change in evaporation and transpiration induced by grazing in long-term overgrazed grassland is likely to be mainly governed by the decrease in living plants, ground litter, and so on [13]. Long-term overgrazing is also likely to alter the structure and function of the grassland ecosystem directly through its effect on plant growth and indirectly via its effects on the soil microenvironment and may have an impact on the global carbon budget, water balance, and nutrient cycling [14, 15].

Generally, PFTs, which can be an indication of ecosystem functions and processes, are more sensitive to abiotic disturbances (such as clipping and grazing) than other ecological levels and processes, e.g. community succession and biodiversity loss [16, 17]. Generally speaking, as leaves are the main functional organ, leaf traits have stable strategies relating to self-protection [18, 19]. Compared with native grassland ecosystems, the specific leaf area (SLA) will increase in response to long-term grazing [20], which is related to improving the ability of the plant to perform photosynthesis [21]. Moreover, the sensitivity of a plant organ in its aboveground portion is greater than in its belowground portion [22]. Root traits have a lag (or stabilizing) effect compared with the soil microenvironment [23]. Different plant species do not follow the same response model to grazing [24]. For example, taller plants have leaves with lower SLA and greater toughness; shorter species with intermediate toughness are more often selected by sheep for consumption. Therefore, short species with high SLA increase with grazing [3]. Our previous research has also reported the different responses of the functional traits of *Leymus chinensis* to long-term overgrazing in meadow steppe land in northern China.

Ecological strategies of grassland plants on a functional trait level (tradeoffs and allometry, for example) are commonly adopted to optimize their growth cycles in adverse habitats [3, 25, 26]. Allometry is defined as the measure and study of growth or size of a part in relation to an entire organism[27]. At the individual level, [28] found that reproductive allometric relationships underwent significant shifts, from a non-allometric relationship in control plots to a fixed allometric relationship following nutrient addition. Also, in paired grazing/non-grazing habitats, the linearized (log—log) slopes of the height—diameter and cover—diameter allometric relationships varied significantly as a result of cattle grazing. Grazing, therefore, can influence plant growth strategies by affecting allometric relationships in plant traits. However, allometric relationships between PFTs and AB have not been well researched in previous studies.

*L*. *chinensis* grassland is one of the most widely distributed types of steppe vegetation in temperate eastern Eurasia[29], and has been traditionally used as rangeland for livestock grazing[30]. Overgrazing is likely to reduce a plant’s individual AB directly through an effect on the phenotype plasticity of different traits and indirectly through the plant traits’ allometric relationships with possible impacts on grassland productivity and biodiversity. However, little is known about the mechanisms responsible, their effects on grassland productivity, and the basic processes involved, i.e. for individual plant biomass decline. Our objective with this paper is to test hypotheses that quantify detected pathways in the decline in individual plant aboveground biomass induced by overgrazing of natural semiarid grassland. We test the hypotheses using *L*. *chinensis* as a model species to compare long-term grazing and grazing-excluded habitats. This species is dominant in the temperate eastern part of the Eurasian steppe lands located in northern China, and has a sensitive response to grazing disturbance. In the present study, we report: (i) AB allocation strategy affected by the variation of the individual size of *L*. *chinensis*; (ii) allometric scaling of AB and PFTs; and (iii) paths of individual *L*. *chinensis* AB decline detected by structural equation modeling (SEM).

## Methods

### Study site

**Fig 1:**
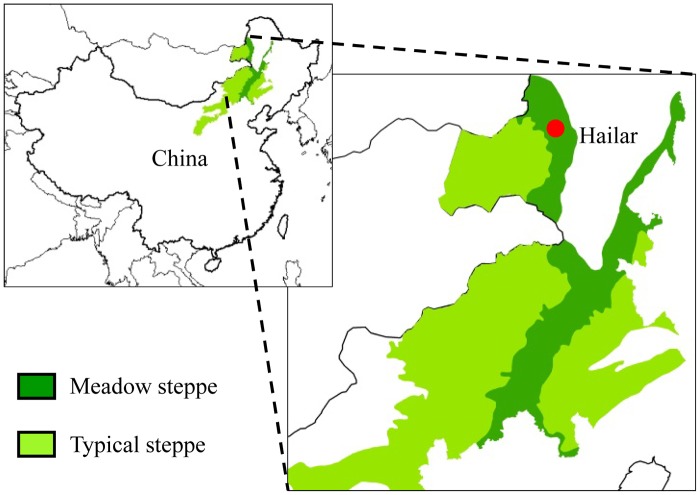
Location map of the study area of experimental plots on *L*. *chinensis* grassland. The meadow steppe and typical steppe in Inner Mongolia regions of China are mainly dominated by *L*. *chinensis*.

A native meadow-steppe grassland was selected in the Hulunber Grassland Ecosystem Observation and Research Station (HGEORS) of the Chinese Academy of Agricultural Sciences (located in the Xiertala farm in the center of the Hulun Buir meadow steppe) (Fig 1). The field site is situated in the easternmost part of the Eurasian steppe in northeastern China (49° 19' N, 120° 02' E, altitude 628m). The mean annual air temperature is between −3 and −1°C. The mean precipitation during the summer (May to September) was approximately 350mm during 1980–2011. The mean length of the growing season is approximately 150 days. The soil in the experimental sites is classified as chestnut (Chinese classification—the soil texture is 42% sand, 35% silt, and 23% clay) and the nutrient availability in the surface layer is low. The bulk density of the soil is 1.37g/cm^3^ and its pH is 7.7 (top 10 cm). The native meadow steppe grassland is dominated by *L*. *chinensis*. Other prevalent grasses include *Stipa baicalensis*, *Cleistogenes squarrosa*, and *Carex duriuscula*[31].

### Experimental design and sampling

Long-term, freely-grazed land and non-grazing (grazing-excluded) land were established in 1997 by HGEORS workers for long-term ecological observation and research in meadow-steppe grassland in the Xiertala farm (Fig 1). The grazing land, measuring more than 200 ha in area, is situated adjacent to the grazing-exclusion land and grazed all year round by more than 1000 sheep and goats for more than 50 years. Therefore, the stocking rate in this region is about 5.0 sheep unit per hectare. It was significant higher than standard stocking rate of local grass-livestock balance (2.5 sheep unit per hectare). Our field studies did not involve endangered or protected species. Most parts of the grazed and grazing-excluded grasslands have similar topographies and altitudes. All the plots used for both grazed and grazing-excluded treatments have the same soil type and similar physiographical conditions (degree of slope, slope direction, topography, and altitude).

Following the principles of replication, randomization, etc., field experiments were established in meadow steppe land dominated by *L*. *chinensis*. Like most chronosequence studies, this set-up was subject to pseudo-replication and space-for-time substitution limitations [30, 32, 33]. Therefore, before our field sampling the middle of the year, five 20 m × 20 m replicated plots were randomly established within the long-term grazed and grazing-excluded regions along a transect line using a paired sampling method in early spring of 2013 (before the growing season). Three 5m × 5m subplots were established in each plot for field investigation and sampling. The subplots were at least 15m apart and did not differ in physiographical conditions. Three 1m×1m quadrats were randomly selected in all of the subplots. On grazing plots, temporary mobile exclosure cages were set up at each sampling point in early April 2013 prior to grazing and before the growing season, which was in order to obtain the whole plant individuals in the sampling year. Field sampling was carried out during 5–10 August 2013, corresponding to the annual peak in standing biomass of the grassland community. Three *L*. *chinensis* individuals were randomly selected from each 1m×1m quadrat. Phenotypic functional traits (Table 1) of the *L*. *chinensis* individuals were measured in a shaded laboratory room after the whole of the aboveground portion of the plant individual had been completely clipped off in the field.

**Table 1:** *L*. *chinensis* plant functional traits used in this research.

Organ	Plant functional trait	Abbreviation	Unit
**Leaf**	**Leaf number**	***LN***	**non-dimensional**
	**Leaf length**	***LL***	**cm**
	**Leaf width**	***LW***	**mm**
	**Leaf length to width ratio**	***LLW***	**cm/mm**
	**Single leaf area**	***SLA***	**cm** ^**2**^
	**Total leaf area**	***TLA***	**cm** ^**2**^
	**Leaf mass per area**	***LMA***	**g/cm** ^**2**^
	**Leaf biomass**	***LB***	**g**
**Stem**	**Stem length**	***SL***	**cm**
	**Stem diameter**	***SD***	**mm**
	**Stem length to diameter ratio**	***SLD***	**cm/mm**
	**Stem biomass**	***SB***	**g**
**Whole aboveground plant**	**Plant height**	***PH***	**cm**
	**Leaf to stem biomass ratio**	***LSB***	**non-dimensional**

Following standard methods, phenotypic traits (such as leaf length, leaf number, stem length, stem width, plant height, etc., as shown in Table 1) were measured using an electronic digital caliper. In addition, leaves were scanned using a digital scanner and their leaf area measured using Adobe Photoshop CS5 software. After measuring the phenotypic traits, the leaves and stems were separately packaged in different paper bags. Then, all of the plant samples were oven-dried at 65°C for at least 48 h before weighing. At the same time the biomass traits were recorded.

### Statistical analysis

The length and width of every leaf on every plant were measured. The averages over all leaves were used to represent the plant’s individual leaf traits. Before carrying out statistical analysis, the functional traits from the three *L*. *chinensis* individuals in a particular quadrat were averaged. Three precipitation utilization efficiency values were calculated by separately dividing the change in functional trait by the mean annual precipitation, growing season precipitation, and precipitation in 2013. Any significant differences in the plant traits obtained from the non-grazed and grazed plots were evaluated using one-way analysis of variance (ANOVA) procedures. The relationships between the plasticity and variability of a PFT and the differences between meadow and typical steppe were fitted to an equation representing an exponential rise to a maximum. Other correlations among the functional traits of *L*. *chinensis* were analyzed using the Pearson method.

The leaf, stem, and plant *L*. *chinensis* functional trait data was transformed logarithmically (base 10). Model Type II regression was used to determine the slope (*a* = scaling exponent) and *y*-intercept (log_10_ *b*, where *b* is the allometric constant) of the log—log linear relationship. The software package *Standardized Major Axis Tests and Routines* [(S)MATR] was also used to determine whether the numerical value of *a* for the log—log plots differ between grazing and non-grazing plots [34]. (S)MATR was also used to provide the Model Type II equivalent of OLS standard analyses of covariance (ANCOVA). The significance level for testing slope heterogeneity was *P* < 0.05 (i.e. the notion of a common slope was rejected if *P* < 0.05). If the compared regressions have common slopes but different *y*-intercepts, then the difference in the *y*-intercepts might lead to a significant difference between the common slope obtained from grazing and non-grazing land and the slope obtained from all the data.

Structural equation modeling was performed to analyze hypotheses that may explain the pathways responsible for the indirect effects of grazing on individual *L*. *chinensis* AB influenced by leaf, stem, and whole-plant functional traits[35]. Experience was used to develop the SEMs by hypothesizing relationships between variables and testing preliminary models. Co-varying factors with non-significant regression weights within the SEMs were not included. The final SEMs were applied to each of the *L*. *chinensis* functional trait indices. Occasionally, it was necessary to alter the relationship between a measured *L*. *chinensis* functional trait and the rest of the model. The utility of each functional trait index within the SEM was compared through a number of measures. These include the power of that model to explain the variation in the *L*. *chinensis* AB, LB and SB (*r* ^2^), measures of model significance and fit (*χ* ^2^, AIC), and the significance of the functional trait variable within the model (significance of regression weights).

All statistical analysis was performed to determine the significance of the treatment means at the *P*<0.05 and *P*<0.01 levels using SPSS 19.0 statistical software. Allometric scaling and SEM were performed using the (S)MATR 2.1 and IBM SPSS AMOS 18 software packages, respectively. Statistical graphs were prepared using Sigmaplot 12.0 software.

## Results

### *L*. *chinensis* individual aboveground biomass in response to overgrazing

**Fig 2:**
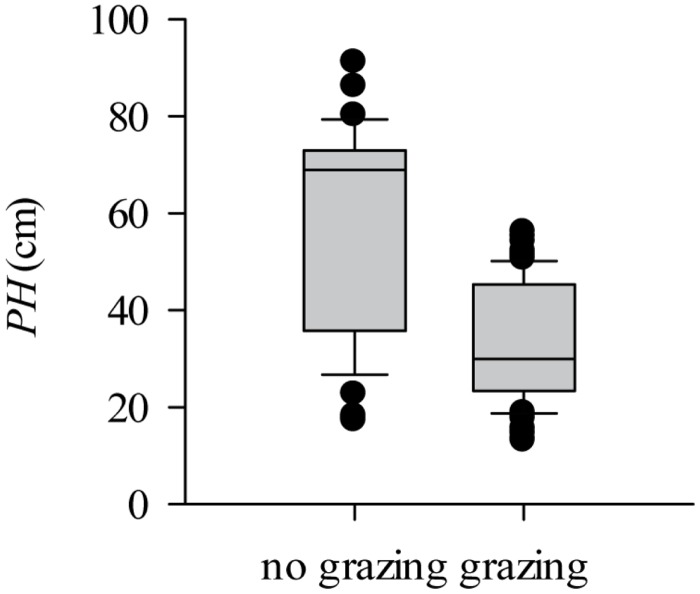
Effect of overgrazing on the height of *L*. *chinensis* individuals in natural grassland. PH was significantly affected by grazing according to ANOVA testing (*F* = 81.97, *P*<0.001).

**Fig 3:**
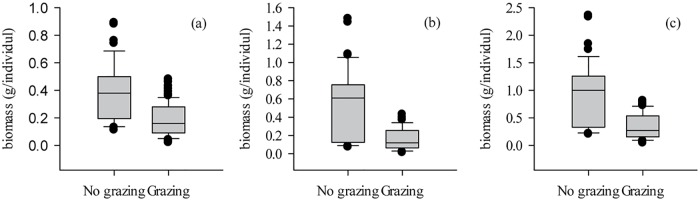
Effect of overgrazing on L. chinensis individual plant aboveground biomass in natural grassland for: (a) the leaf biomass per individual (ANOVA results: F = 65.06, *P*<0.001), (b) the stem biomass per individual (*F* = 89.38, *P*<0.001), and (c) the aboveground biomass per individual, including leaf and stem (*F* = 83.78, *P*<0.001).

Overgrazing caused the plant height (PH) of individual *L*. *chinensis* to decrease significantly (by 42.57%, *P*<0.001) compared to those grown with no grazing (Fig 2). The significant miniaturization of *L*. *chinensis* individuals shows that they were affected by overgrazing (*P*<0.01, S1 Fig). Within this background of morphological miniaturization, the leaf biomass, stem biomass, and the whole-plant aboveground biomass in the grazed plots were highly significantly reduced by 52.97%, 69.88%, and 62.69% compared to that in non-grazed plots, respectively (*P*<0.001, Fig 3).

### Biomass allocation strategy affected by the variation of individual size

**Fig 4:**
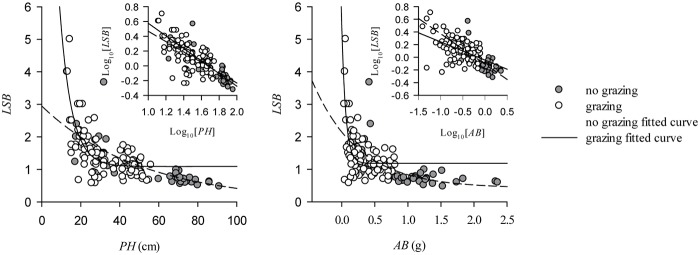
Effect on aboveground biomass allocations of the overgrazing-induced variation in the size of L. chinensis individuals. The leaf to stem biomass ratio of *L*. *chinensis* individuals is fitted to an exponential decay relation as a function of PH and AB. In the non-grazing group, the leaf to stem biomass ratio is significantly affected by PH (*R* = -0.78, *P*<0.001) and AB (*R* = -0.67, *P*<0.001). The leaf to stem biomass is also significantly affected by PH (*R* = -0.75, *P*<0.001) and AB (*R* = -0.64, *P*<0.001) in grazed plots. Overall, PH (*R* = -0.73, *P*<0.001) and AB (*R* = -0.62, *P*<0.001) significantly influence leaf to stem biomass (insets). The gray arrows in the figures show the shifts in the slopes of the exponential decay relations as a result of overgrazing.

**Fig 5:**
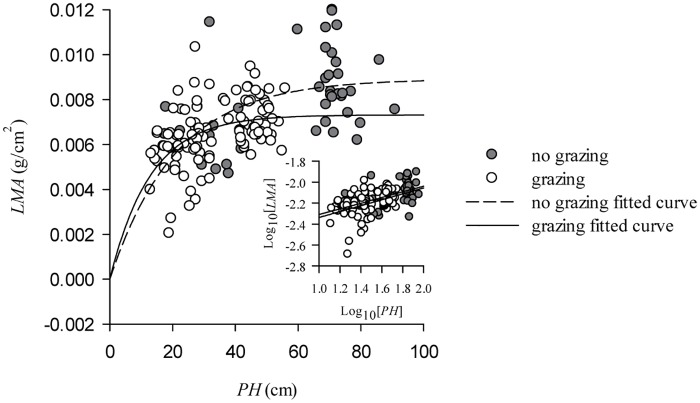
Effect on LMA of the variation in the size of L. chinensis individuals induced by grazing. The relationship between LMA and PH was fitted to an equation representing an exponential rise to a maximum for both the non-grazing (*R* = 0.42, *P*<0.001) and grazing groups (*R* = 0.45, *P*<0.001). LMA was also significantly affected by PH (*R* = 0.51, *P*<0.001) for the combined data (inset).

Leaf allocation of *L*. *chinensis* aboveground biomass significantly increased with decreasing individual size as a result of overgrazing (S2 Fig). The leaf to stem biomass ratio is significantly negatively correlated with PH and AB in the patterns of exponential decay found not only for non-grazed plots but also for grazed plots (*P*<0.001). Importantly, the leaf to stem biomass ratio is more sensitive to variation in the individual size in grazed plots compared to non-grazed plots (Fig 4). Leaf mass per unit area (LMA), however, is significantly positively correlated with PH in the patterns of exponential rise to maximum (*P*<0.001). Also, overgrazing not only decreases the maximum LMA in the equation but also causes a decrease in LMA on the whole (Fig 5; S3 Fig).

### Allometric scaling of aboveground biomass and PFT

**Fig 6:**
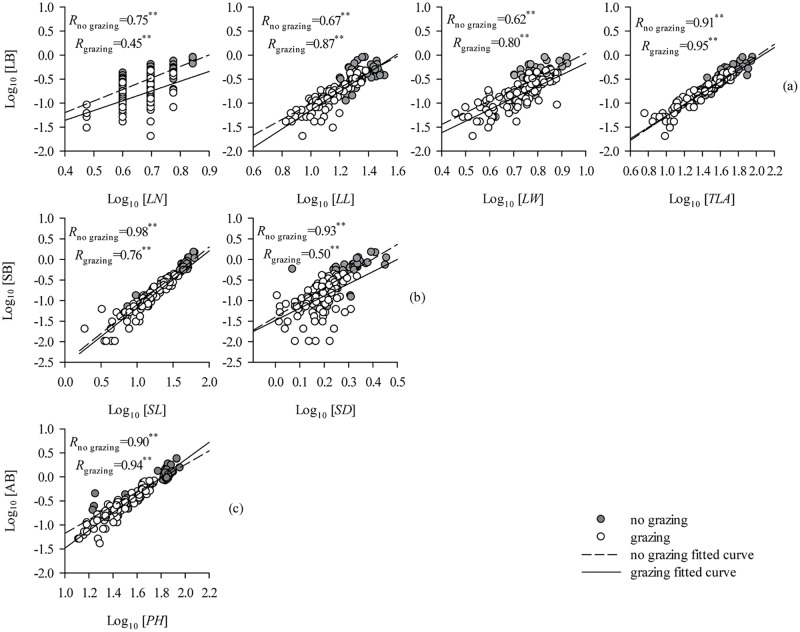
Allometric scaling of PFT and biomass in: (a) the leaves, (b) the stems, and (c) the whole *L*. *chinensis* plant. The figures show the standardized major axis regressions (see also Table 1). The relationships between PFT and biomass in the leaves, stems, and whole plant are best described using a linear-equation-based SMA (*P*<0.01).

Overall, the leaf, stem, and whole-plant aboveground biomass, are highly significantly correlated with various functional traits (*P*<0.01, Table 2). The log-log scaling relationships for biomass and morphological traits show that significant allometric relationships exist, not only in the non-grazed grassland, but also in the grazed grassland (*P*<0.01, Fig 6 and Table 3). The slopes of the allometric equations for [LB *vs*. LN], [LB *vs*. LW], and [AB *vs*. PH], which show significant heterogeneity in the non-grazing and grazing groups, are significantly influenced by overgrazing (*P*<0.05, Table 3). In contrast, standardized major axis (SMA) tests for common slopes reveal no significant difference in the slopes of the bivariate relationships [LB *vs*. LL], [LB *vs*. TLA], [SB *vs*. SL], and [SB *vs*. SD] exhibited by non-grazing and grazing groups (*P*>0.05, Table 3). There were, however, significant shifts along the common slope of four of the bivariate relationships (Table 3). Also, the allometric scaling of LB and SB was significantly affected by grazing (S4 Fig).

**Table 2:** Relationships between aboveground biomass and the PFTs of *L*. *chinensis* individuals.

Organ	PFT	Combined	Non-grazing	Grazing
**Leaf**	**LN**	0.60**	0.75**	0.35**
	**LL**	0.78**	0.56**	0.92**
	**LW**	0.66**	0.62**	0.80**
	**LLW**	0.51**	0.27**	0.42**
	**SLA**	0.79**	0.62**	0.93**
	**TLA**	0.90**	0.86**	0.96**
	**LMA**	0.67**	0.66**	0.55**
	**LB**	0.97**	0.97**	0.97**
**Stem**	**SL**	0.94**	0.91**	0.90**
	**SD**	0.80**	0.80**	0.59**
	**SLD**	0.80**	0.73**	0.78**
	**SB**	0.99**	0.99**	0.97**
**Whole aboveground plant**	**PH**	0.92**	0.86**	0.94**
	**LSB**	-0.52**	-0.61**	-0.46**

All the correlations relate to a linear fit and the correlation coefficients are all significant at the 0.01 level (** in the table).

**Table 3:** Standardized major axis regression slopes and confidence intervals (CIs) for log-log transformed relationships between aboveground biomass and PFTs of *L*. *chinensis* for non-grazing and long-term overgrazing groups.

Bivariate relationship *(Y- vs*. *X*-axis*)*	Group	Slope			Heterogeneity of slopes			Common slope *Y*-axis intercept	Shift along a common slope?
		Value	CI	*P*	Common slope	CI	*P*		
**LB *vs*. LN**	**non-grazing**	**3.22**	**(2.63, 3.94)**	**<0.001**	**3.90**		**0.02**		
	**grazing**	**4.49**	**(3.77, .33)**	**<0.001**					
**LB *vs*. LL**	**non-grazing**	**2.46**	**(1.96, 3.09)**	**<0.001**	**2.27**	**(2.08, 2.48)**	**0.42**	**-3.49**	**yes**
	**grazing**	**2.24**	**(2.03, 2.46)**	**<0.001**				**-3.47**	
**LB *vs*. LW**	**non-grazing**	**3.97**	**(3.13, 5.04)**	**<0.001**	**3.17**		**0.03**		
	**grazing**	**3.01**	**(2.67, 3.38)**	**<0.001**					
**LB *vs*. TLA**	**non-grazing**	**1.38**	**(1.21, 1.57)**	**<0.001**	**1.28**	**(1.21, 1.35)**	**0.20**	**-2.57**	**yes**
	**grazing**	**1.25**	**(1.18, 1.33)**	**<0.001**				**-2.58**	
**SB *vs*. SL**	**non-grazing**	**1.43**	**(1.35, 1.52)**	**<0.001**	**1.45**	**(1.39, 1.53)**	**0.34**	**-2.58**	**yes**
	**grazing**	**1.50**	**(1.39, 1.61)**	**<0.001**				**-2.65**	
**SB *vs*. SD**	**non-grazing**	**4.68**	**(3.83, 5.71)**	**<0.001**	**5.37**	**(4.72, 6.14)**	**0.07**	**-1.91**	**yes**
	**grazing**	**5.94**	**(5.02, 7.03)**	**<0.001**				**-1.92**	
**AB *vs*. PH**	**non-grazing**	**1.58**	**(1.39, 1.81)**	**<0.001**	**1.88**		**0.01**		
	**grazing**	**1.96**	**(1.83, 2.09)**	**<0.001**					

The 95% CIs for the SMA and common slopes are shown in parentheses. (SMA regression intercepts and their CIs are given in S1 Table.) In several of the bivariate cases, SMA tests for common slopes reveal no significant differences between the two treatment groups (i.e. *P*>0.05). In such cases, common slopes for the bivariate relationships are shown with their CIs. Where there was a significant difference in elevations (i.e. *Y*-axis intercepts) of the common-slope SMA regressions, the values of the *Y*-axis intercepts are provided. Significant shifts along a common slope are also indicated.

### Paths of *L*. *chinensis* individual aboveground biomass decline

**Fig 7:**
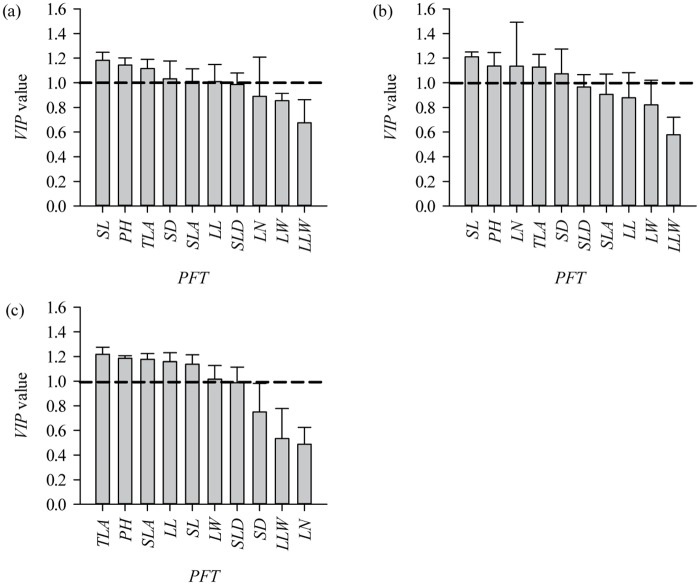
VIP values (mean + SD) of PFTs of *L*. *chinensis* individuals with respect to AB in: (a) non-grazing, (b) grazing, and (c) combined plots. The dashed line in each figure represents a VIP value of 1.

Different PFTs play a different role in the decline of individual *L*. *chinensis* aboveground biomass as a result of overgrazing. SL, PH, TLA, etc. play a more important role (VIP>1.00) than other PFTs on the whole (Fig 7; Table 4). Also, stem biomass has a higher contribution rate to the variation of individual *L*. *chinensis* AB than leaf (Fig 6).

**Table 4:** The proportion of the AB variation in *L*. *chinensis* individuals explainable using morphological PFTs.

Organ	PFT	Combined	Non-grazing	Grazing
**Leaf**	**LN**	**0.09**	**0.12**	**0.05**
	**LL**	**0.10**	**0.09**	**0.12**
	**LW**	**0.09**	**0.08**	**0.11**
	**LLW**	**0.07**	**0.06**	**0.06**
	**SLA**	**0.10**	**0.09**	**0.12**
	**TLA**	**0.11**	**0.11**	**0.13**
**Stem**	**SL**	**0.12**	**0.12**	**0.12**
	**SD**	**0.10**	**0.11**	**0.08**
	**SLD**	**0.10**	**0.10**	**0.10**
**Whole plant**	**PH**	**0.12**	**0.12**	**0.12**

In each of the three groups (combined, non-grazing, and grazing), the proportions all add up to 1.00.

**Fig 8:**
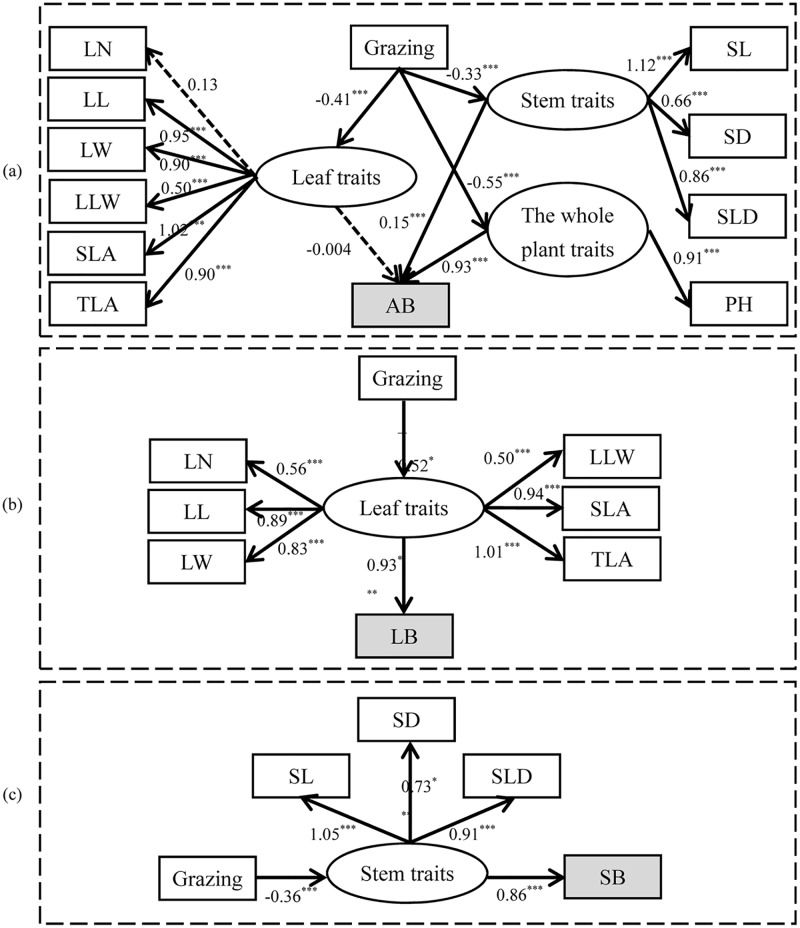
Final results of the SEM analysis of the effects of overgrazing on: (a) AB, (b) LB, and (c) SB of *L*. *chinensis* individuals via PFT pathways. Boxes and ellipses denote the observed and latent variables included in the models, respectively. Solid arrows denote the directions and effects that are significant (*P*<0.001, marked *** in the figure); dashed arrows represent the directions and effects that are not significant (*P*>0.05). Values associated with arrows represent standardized path coefficients. Estimates of the regression weights in the SEM analysis are given in S2 Table, S3 Table, and S4 Table.

Direct and indirect paths of individual *L*. *chinensis* aboveground biomass decline induced by grazing were fitted using SEM (Fig 8; S2, S3, and S4 Tables). Grazing indirectly affects AB by a ‘bottom-up’ effect of PFTs. Plant height and stem traits are the most important pathways determining AB decline (*P*<0.001), while the leaf-trait pathway is not significantly important (*P*>0.05, Fig 8a). On a functional-organ scale, grazing negatively affects LB and SB of *L*. *chinensis* individuals indirectly via morphological PFT pathways (*P*<0.001, Fig 8b and 8c).

## Discussion

### The role of individual plants in grazing-induced grassland productivity decline

Natural grassland ecosystem productivity, which relies on individual plant biomass to some extent, is the most important function of the grassland ecosystem [36]. The fall in grassland productivity due to pressure from human activity and other global changes affects ecosystems globally. To date, there have been many studies reporting the mechanism of productivity decline from the point of view of biodiversity loss [37]. However, little is known about how plant traits change in response to long-term grazing, an important process that influences grassland productivity [38]. Our results demonstrate that grazing has significant effects on a plant’s leaf and stem functional traits. As a result, individual plants became smaller compared with the same species in the un-grazed grassland community. Furthermore, the diminution of phenotypic traits derived from the individual plant aboveground biomass declined in habitats undergoing grazing and clipping. Thus, the impact on phenotypic plasticity of grassland plants caused by disturbances is a key process and mechanism leading to decline of grassland productivity.

Functional traits which link to ecosystem functions are basic elements that adapt to grazing [39]. In our results, due to the impact of long-term grazing, leaf number, leaf length, leaf width, leaf area, stem length, stem diameter, plant height, etc. in semi-arid grassland ecosystems decreased significantly compared to plants in non-grazed habitats. Some studies have found that not all grassland plant species have the same mode of phenotypic plasticity reaction. There were distinct differences in the responses of grazing-susceptible and grazing-resistant species to abiotic disturbances in long-term heterogeneous habitats [24]. More remarkably, *L*. *chinensis*, a dominant species in Inner Mongolia’s grasslands, was found to be more susceptible in the grassland population. The trait of miniaturization in *L*. *chinensis* would significantly affect the structure and function of a grassland ecosystem. So cascade reactions in grasslands happen at different levels, from functional traits to individuals, species, population, and ecosystem [40, 41].

### Biomass allocation strategies in response to overgrazing

Our results demonstrate that the slopes of the exponential decay equation for the relationships between the leaf to stem biomass ratio and PH and AB clearly change as a result of overgrazing. The result suggests that an *L*. *chinensis* individual will invest more of its photosynthetic products in leaves than stems during the process of plant miniaturization. Generally speaking, leaves are one of the most important organs of a plant required to achieve the functions of photosynthesis, transpiration, nutrient utilization, and so on. Due to the pressure of long-term overgrazing, individual plant photosynthesis is limited by herbivore foraging [42, 43]. It may be that as a result of the grazing-induced increase in leaf to stem biomass ratio, the plant can enhance its ability to carry out photosynthesis in the presence of herbivore foraging [44]. Furthermore, our results indicating increased leaf to stem biomass with individual plant miniaturization may be a potential mechanism for the plant to adapt to grazing-induced changes in plant-soil feedback.

Moreover, LMA is an effective index which is correlated with a leaf’s photosynthetic capacity and can be used to evaluate a leaf’s economic strategy to adapt to a heterogeneous habitat. Zheng et al. (2011) indicated that two contrasting dominant species, *L*. *chinensis* and *Cleistogenes squarrosa*, have different responses in their plant functional traits to grazing caused by trait trade-offs in different habitats. For example, under grazed conditions, *L*. *chinensis* adopts a high nitrogen content and net photosynthetic rate in wet years but low nitrogen content and net photosynthetic rate in dry years. The trait trade-off of *C*. *squarrosa* is characterized by a high specific leaf area and net photosynthetic rate in dry years *vs*. low specific leaf area and net photosynthetic rate in wet years. In this study, we have demonstrated that LMA (reciprocal calculation with specific leaf area) varies significantly with decreasing PH in the exponential rise to maximum pattern. Interestingly, overgrazing not only decreased the maximum LMA in the equation but also decreased the LMA on the whole. Accordingly, *L*. *chinensis* will invest limited photosynthetic resources to maximizing leaf area in order to improve the plant’s capacity to utilize light.

### Allometric scaling of aboveground biomass

We have shown that the plant’s weight traits (LB, SB, and AB) are more sensitive to grazing than its morphological traits. This result demonstrates that different PFTs respond asymmetrically during effective adaption to long-term overgrazing. Enlightened by our other results, it seems that mechanisms related to allometric scaling of *L*. *chinensis* aboveground biomass may potentially explain this scientific phenomenon. In this study, using SMA tests, we found that significant allometric relationships exist between biomass and morphological traits not only in the non-grazed but also the grazed grassland. Accordingly, the plants invested fewer biomass resources per unit PH, LL, SL, etc. with the decrease in the plant’s individual size. Also, the slopes of the allometric equations for AB and PH were significantly heterogeneous in the non-grazing and grazing groups. Grazing increased the slopes of the AB and PH plots in comparison with non-grazing plots. The ability of the aboveground biomass to respond per unit plant height was enhanced to cope with the grazing-induced miniaturization of *L*. *chinensis* individuals.

Similarly, some previous studies have already shown that plants can allocate resources differently in direct response to grazing and that grazing-induced legacy effects in the soil can increase nitrogen and biomass allocation to shoots [44]. In this study, we have shown that there are different ways aboveground biomass and its components respond to grazing and non-grazing. As a mechanism of adaption to grazing, therefore, the aboveground biomass (and that belowground) and their compositions are adjusted in the dry-matter distribution strategy. This may be rooted in the direct response of the plant to grazing.

### Mechanisms of grazing-induced individual plant AB decline at the PFT scale

Our results indicate that overgrazing indirectly affects the plant’s aboveground biomass via the response of the PFTs to grazing. Although we demonstrated that leaf, stem, and whole-plant aboveground biomass are highly and significantly correlated with various functional traits on the whole, we also found that different kinds of PFT, i.e. morphological or biomass, played different roles in the decline of individual *L*. *chinensis* AB through overgrazing. For example, SL, PH, TLA, etc., are key traits in driving AB decline in the presence of long-term overgrazing. Furthermore, our results demonstrate that grazing indirectly affects AB by a PFT ‘bottom-up’ effect. Also, whole-plant and stem traits play more important roles in AB decline than leaf traits. This implies that there were tradeoffs among the different traits in response to grazing.

## Conclusion

In conclusion, our results indicated that a highly significant decline in the individual height and biomass (leaf, stem, and the whole plant) of *L*. *chinensis* as a result of overgrazing. Biomass allocation and leaf mass per unit area were significantly affected by the variation in individual size. Grazing clearly enhanced the sensitivity of the leaf-to-stem biomass ratio in response to variation in individual size. Moreover, we found that the biomass in the leaves, stems, and the plant as a whole had highly significant allometric scaling with various functional traits. Also, the slopes of the allometric equations of these relationships were significantly altered by grazing. Therefore, a clear implication of this is that grazing promotes an asymmetrical response of different plant functional traits to variation in individual plant size, which influences biomass indirectly. Furthermore, we detected paths of individual AB decline in *L*. *chinensis* induced by grazing by fitting to a structural equation model. These results indicate that grazing causes AB decline primarily through a ‘bottom-up’ effect on plant height and stem traits. However, leaf traits, via the process of allometric scaling, affect plant AB indirectly.

## Supporting Information

S1 Fig The response sensitivities of different *L*. *chinensis* PFTs to grazing sorted according to plasticity index (PI). The PI value, which quantifies the degree of response of a PFT to grazing, was calculated using PI = (PFT_non-grazing_— PFT_grazing_)/PFT_non-grazing_. The differences in the PFTs in non-grazed and grazed grassland were analyzed using ANOVA tests; in the figure, ** and *** represent significant differences at the 0.01 and 0.001 levels, respectively.
(TIF)

S2 Fig Effect of overgrazing on the leaf to stem biomass ratio in *L*. *chinensis* individuals in natural grassland. LSB is significantly affected by grazing as tested by ANOVA (*F* = 11.78, *P* = 0.001).
(TIF)

S3 Fig Effect of overgrazing on the LMA of *L*. *chinensis* individuals in natural grassland.LMA is significantly affected by grazing as tested by ANOVA (*F* = 25.09, *P*<0.001). (TIF)

S4 Fig Allometric scaling of the LB and SB of *L*. *chinensis*. The LB and SB (g/individual) values were first log_10_-transformed. The relationships between LB and SB in non-grazing and grazing plots are best described using a linear function-based SMA (non-grazing plots: *R* ^2^ = 0.85, 95% CI for the slope = 1.43–1.82, *P*<0.001; grazing plots: *R* ^2^ = 0.81, 95% CI for the slope = 1.21–1.44, *P*<0.001). The slopes of the allometric equations for [LB *vs*. SB], which show significant heterogeneity in non-grazing and grazing groups, are significantly influenced by overgrazing (common slope = 1.41>1.00, *P* = 0.001) as analyzed by SMA regression.
(TIF)

S1 Table Standardized major axis regression intercepts and their CIs for log-log transformed relationships comparing aboveground biomass and the PFTs of *L*. *chinensis* in non-grazing and long-term overgrazing groups. (XLSX)

S2 Table Estimates of regression weights in the final model results from SEM analysis of the overgrazing effects on AB via PFT pathways. Abbreviations: SE = standard error; CR = critical ratio; “–” represents a regression weight fixed at 1.00, i.e. not estimated.
(XLSX)

S3 Table Estimates of regression weights in the final model results from SEM analysis of the overgrazing effects on LB via PFT pathways. The annotations are as in S2 Table.
(XLSX)

S4 Table Estimates of regression weights in the final model results from SEM analysis of the overgrazing effects on SB via PFT pathways. The annotations are as in S2 Table.
(XLSX)
