# Supplementary figures and images for: Pathways of Leymus chinensis Individual Aboveground Biomass Decline in Natural Semiarid Grassland Induced by Overgrazing: A Study at the Plant Functional Trait Scale
Source: PLoS One. 2015 May 5;10(5):e0124443. doi: 10.1371/journal.pone.0124443 (PMC4420280; doi:10.1371/journal.pone.0124443)

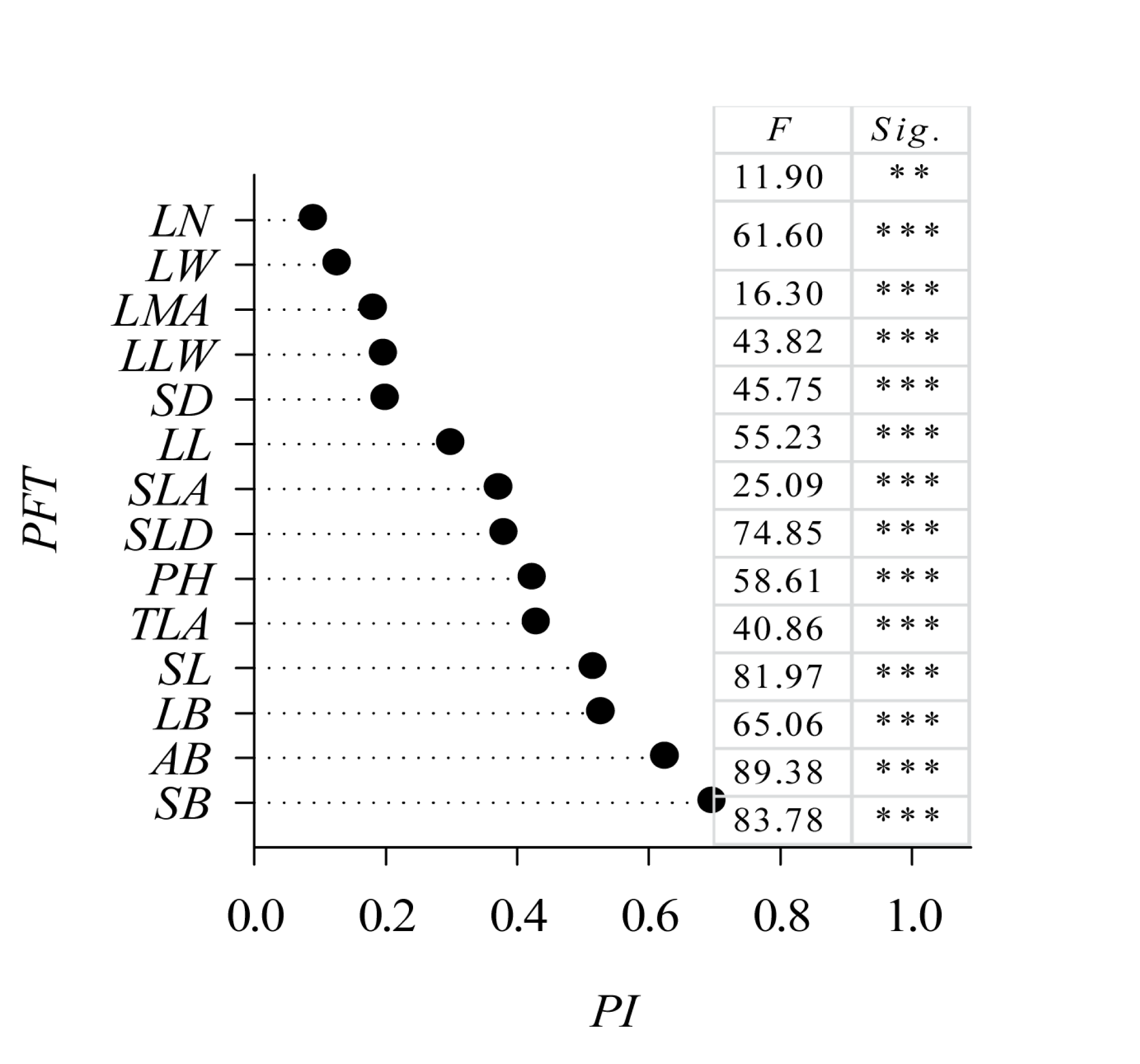

Supplement: S1 Fig — The PI value, which quantifies the degree of response of a PFT to grazing, was calculated using PI = (PFTnon-grazing— PFTgrazing)/PFTnon-grazing. The differences in the PFTs in non-grazed and grazed grassland were analyzed using ANOVA tests; in the figure, ** and *** represent significant differences at the 0.01 and 0.001 levels, respectively. (TIF) [file pone.0124443.s001.tif]

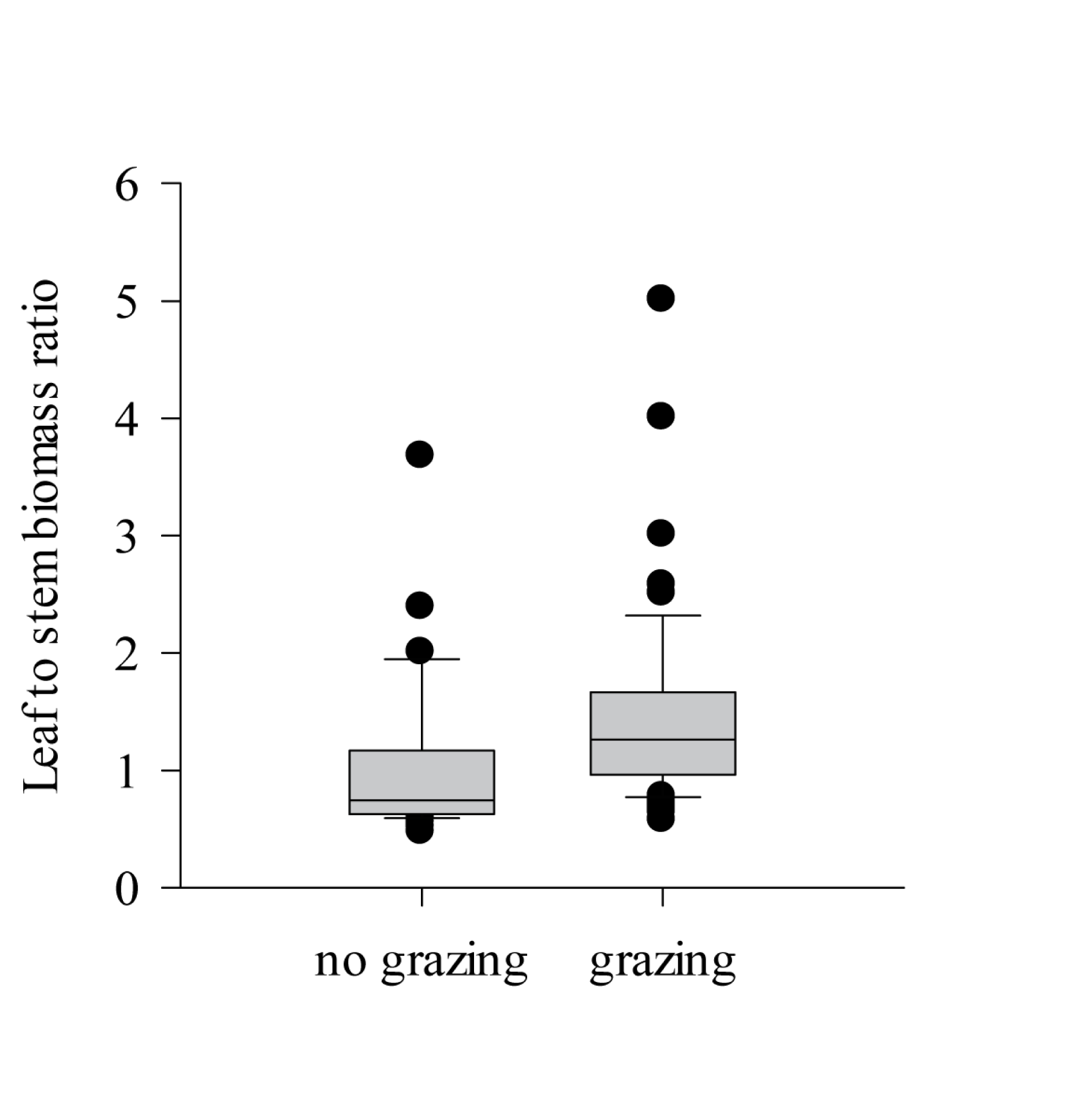

Supplement: S2 Fig — LSB is significantly affected by grazing as tested by ANOVA (F = 11.78, P = 0.001). (TIF) [file pone.0124443.s002.tif]

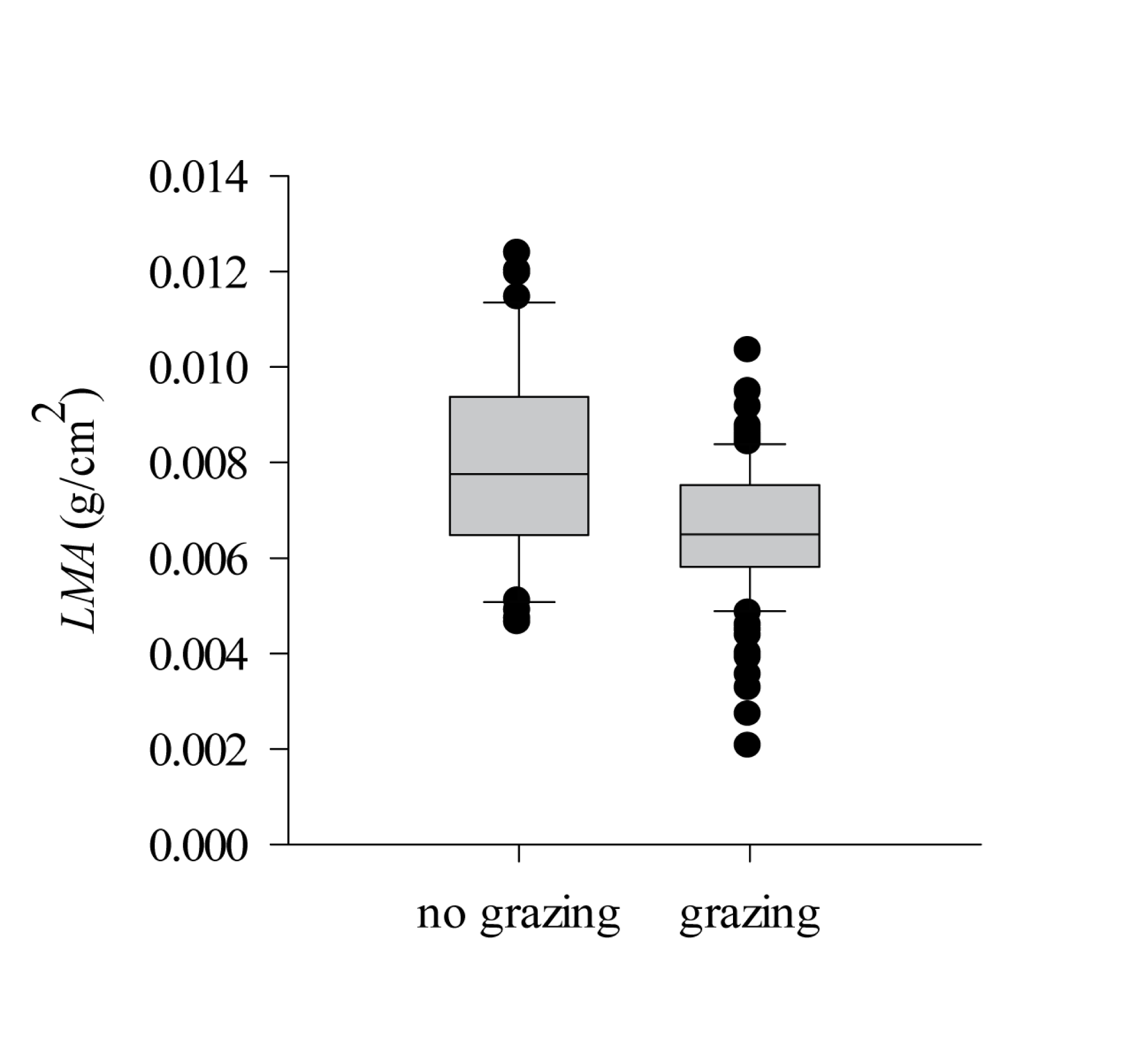

Supplement: S3 Fig — (TIF) [file pone.0124443.s003.tif]

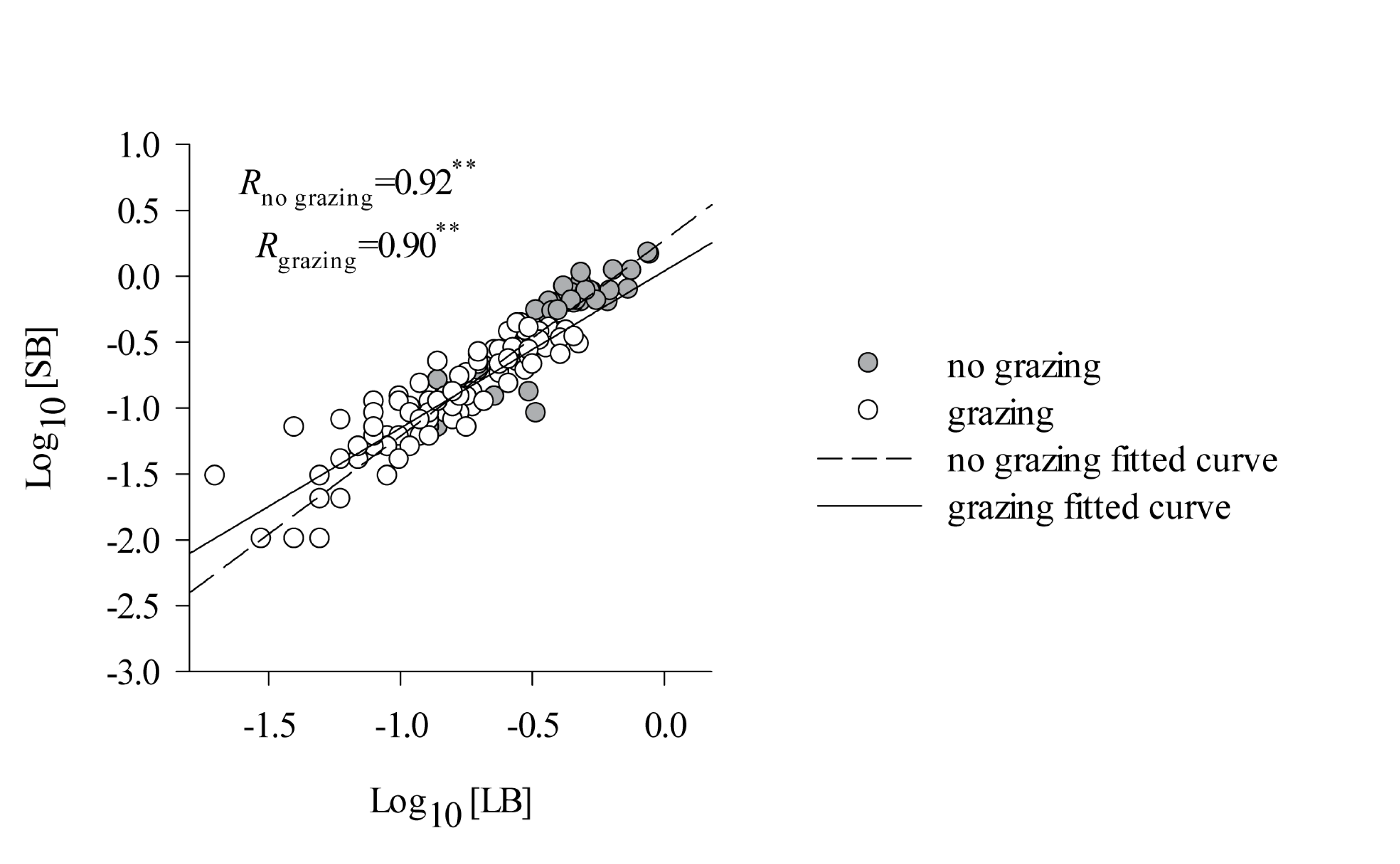

Supplement: S4 Fig — The relationships between LB and SB in non-grazing and grazing plots are best described using a linear function-based SMA (non-grazing plots: R 2 = 0.85, 95% CI for the slope = 1.43–1.82, P<0.001; grazing plots: R 2 = 0.81, 95% CI for the slope = 1.21–1.44, P<0.001). The slopes of the allometric equations for [LB vs. SB], which show significant heterogeneity in non-grazing and grazing groups, are significantly influenced by overgrazing (common slope = 1.41>1.00, P = 0.001) as analyzed by SMA regression. (TIF) [file pone.0124443.s004.tif]
